# The role of interconnected hub neurons in cortical dynamics

**DOI:** 10.1186/1471-2202-15-S1-P158

**Published:** 2014-07-21

**Authors:** Hesam Setareh, Moritz Deger, Wulfram Gerstner

**Affiliations:** 1 School of Life Sciences, Brain Mind Institute and School of Computer and Communication Sciences, École polytechnique fédérale de Lausanne, 1015 Lausanne EPFL, Switzerland

## 

The structure of synaptic connectivity plays an important role in information processing and dynamics of neuronal microcircuits. Previous work has shown that cortical microcircuits contain non-random features of the network structure [1], and that these affect neuronal dynamics [2]. Earlier models of non-random network structure proposed local correlations in synaptic weight or connection number (degree) [3,4]. In such network models, there are neurons receiving stronger synaptic weights or higher numbers of synapses compared to other neurons. Here, we refer to the former neuron type as hub neuron, or simply hub. In other words, a hub receives strong synapses, but not necessarily a higher number of synapses. Importantly, a hub results from the structure of the network and not from differences in neuron parameters. Here we introduce the network feature of connectedness of hub neurons. We show that an elevated connection probability between hubs affects various aspects of network activity, ranging from spontaneous oscillations to the response of cortical populations to stimulation.

A subpopulation of connected hubs can be analyzed using common mean-field methods. This analysis reveals two stable fixed points of the spiking activity, one at a low firing rate and another one at a high firing rate.

**Figure 1:**
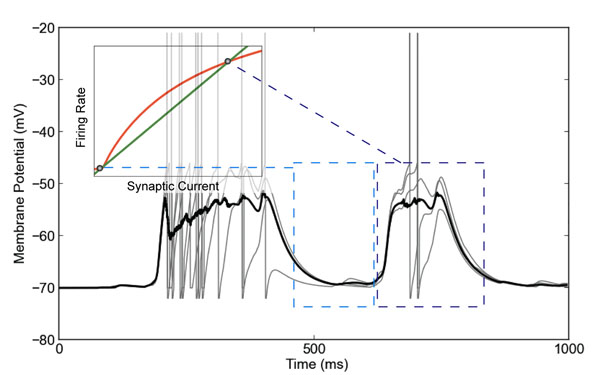
Neuronal populations with connected hubs show up/down state oscillations (simulation). Membrane potential of individual neurons (gray) and average over all neurons (black). Inset indicates self-consistent rate analysis. The two rate fixed points are associated with the up and down states.

We first model a single layer of a column of rodent barrel cortex. The subpopulation of connected hubs switches between the two fixed points and generates up-state/down-state oscillations (see Figure 1), and thus acts as the “heart” of the oscillator. Furthermore, we demonstrate that the different characteristics of cortical layer responses in optogenetic stimulation experiments [5] can be explained by the absence or presence of connected hubs. Finally, we extend the system to contain several connected hubs subpopulations. Such multi-heart oscillators generate irregular oscillations, reminiscent of spontaneous cortical activity.
